# A 15-year-old girl with pancytopenia and congenital defects

**DOI:** 10.4103/0256-4947.55315

**Published:** 2009

**Authors:** Ahmed M. AlSuliman, Kafiah Al Qadaiub

**Affiliations:** From the Medical Department, King Fahad Hospital, Hofuf, Saudi Arabia

A 15-year-old girl was referred to our hospital because of lethargy, palpitation and headache. Physical examination revealed a girl with short stature, café-au-lait spots, and left thumb abnormality. The hematologic parameters of the patient included hemoglobin 5.5 g/dL, WBC 2.96×10^9^/L, platelets 38×10^9^/L, and mean corpuscular volume 100 fL. Radiologic examination of the hands and abdomen revealed abnormal findings that gave a clue to the diagnosis.

1. What is the abnormal finding on this plain radiograph of the hands (Figure 1)?

**Figure 1:**
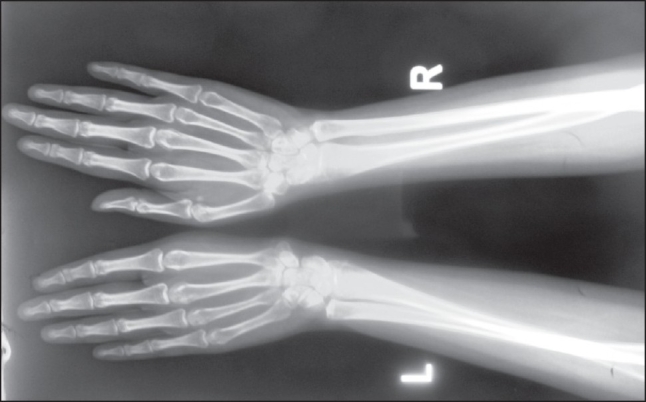
Plain radiograph of the hands.
2. What are the abnormal findings on this abdominal CT scan (Figure 2)?

**Figure 2:**
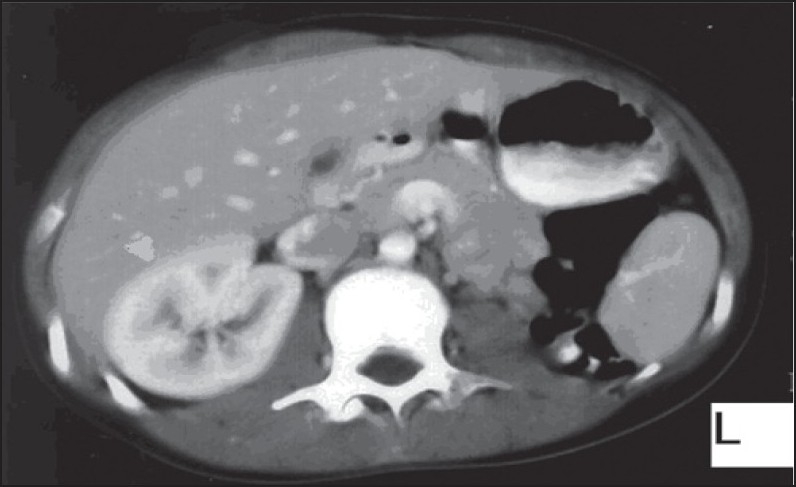
Abdominal CT scan.
3. What's your diagnosis?

FOR THE ANSWER, VISIT:

http://www.saudiannals.net

## Diagnosis: Fanconi anemia

The abnormal finding in Figure 1 is the absence of the left thumb. In Figure 2, the abnormal findings are the absence of the left kidney and compensatory hypertrophic large right kidney. The diagn nosis is Fanconi anemia with short stature, café-au-lait spot, pancytopenia, absence of the left thumb, and absence of the left kidney.

## DISCUSSION

Fanconi anemia (FA) is an autosomal recessive disease, characterized by congenital abnormalities,^1^ in addition to defective hematopoiesis, and a high risk of developing acute myeloid leukemia and certain solid tumors.^2^,^3^

Congenital abnormalities include skin pigmentation and/or café au lait spots, short stature, malformation of the skeleton (microcephaly, spina bifida, scoliosis, absent radii or thumbs). Congenital malformations of the thumbs are variable and often bilateral.^4^ Abnormal male gonads formation, head, eyes, ear, genitourinary, gastrointestinal tract, cardiopulmonary, central nervous system can occur.^1^,^5^–^7^

The most important clinical features of Fanconi anemia are hematological. Fanconi anemia is the commonest type of inherited bone marrow failure syndrome and the incidences of aplastic anemia, myelodysplastic syndrome (MDS), and acute myeloid leukemia (AML).^8^,^9^ Cells from Fanconi anemia patients show an abnormally high frequency of spontaneous chromosomal breakage and the diagnostic test is elevated breakage after incubation of peripheral blood lymphocytes with DNA cross-linking diepoxybutane (DEB test).^10^,^11^

Allogeneic stem cell transplantation from an HLA-matched sibling donor is the only curative therapy, and mild conditioning regimes are used because of the sensitivity of patient's cell to DNA damage.^12^,^13^
